# A new humanized *in vivo* model of *KIT* D816V^+^ advanced systemic mastocytosis monitored using a secreted luciferase

**DOI:** 10.18632/oncotarget.12824

**Published:** 2016-10-22

**Authors:** Siham Bibi, Yanyan Zhang, Caroline Hugonin, Mallorie Depond Mangean, Liang He, Ghaith Wedeh, Jean-Marie Launay, Sjoerd Van Rijn, Thomas Würdinger, Fawzia Louache, Michel Arock

**Affiliations:** ^1^ Molecular and Cellular Oncology Research Group, LBPA CNRS UMR 8113, Ecole Normale Supérieure de Cachan, Cachan, France; ^2^ INSERM Unit U1170, Hématopoïèse normale et pathologique, Gustave Roussy Campus, Université Paris Sud Villejuif, France; ^3^ Laboratoire de Biochimie et Biologie Moléculaire, Inserm U942, Hôpital Lariboisière, AP-HP, Université Paris Diderot - Paris VII Paris, France; ^4^ Neuro-oncology Research Group, Department of Neurosurgery, Cancer Center Amsterdam, VU University Medical Center, Amsterdam, The Netherland; ^5^ Neuroscience Center, Department of Neurology, Massachussetts General Hospital and Neuroscience Program, Harvard Medical School, Boston, MA, USA; ^6^ Laboratoire Central d'Hématologie, Groupe Hospitalier Pitié-Salpêtrière, AP-HP, Université Pierre et Marie Curie (UPMC) Paris VI, Paris, France

**Keywords:** KIT D816V mutant, ROSA^KIT D816V^ cell line, gluc reporter, NSG mice, advanced systemic mastocytosis

## Abstract

Systemic mastocytosis are rare neoplasms characterized by accumulation of mast cells in at least one internal organ. The majority of systemic mastocytosis patients carry *KIT* D816V mutation, which activates constitutively the KIT receptor. Patient with advanced forms of systemic mastocytosis, such as aggressive systemic mastocytosis or mast cell leukemia, are poorly treated to date. Unfortunately, the lack of *in vivo* models reflecting *KIT* D816V^+^ advanced disease hampers pathophysiological studies and preclinical development of new therapies for such patients. Here, we describe a new *in vivo* model of *KIT* D816V^+^ advanced systemic mastocytosis developed by transplantation of the human ROSA^KIT D816V-Gluc^ mast cell line in NOD-SCID IL-2R g^−/−^ mice, using *Gaussia princeps* luciferase as a reporter. Intravenous injection of ROSA^KIT D816V-Gluc^ cells led, in 4 weeks, to engraftment in all injected primary recipient mice. Engrafted cells were found at high levels in bone marrow, and at lower levels in spleen, liver and peripheral blood. Disease progression was easily monitored by repeated quantification of *Gaussia princeps* luciferase activity in peripheral blood. This quantification evidenced a linear relationship between the number of cells injected and the neoplastic mast cell burden in mice. Interestingly, the secondary transplantation of ROSA^KIT D816V-Gluc^ cells increased their engraftment capability. To conclude, this new *in vivo* model mimics at the best the features of human *KIT* D816V^+^ advanced systemic mastocytosis. In addition, it is a unique and convenient tool to study the kinetics of the disease and the potential *in vivo* activity of new drugs targeting neoplastic mast cells.

## INTRODUCTION

Human mast cells (MCs) are non-circulating, tissue-resident cells derived from CD34^+^ hematopoietic stem cells in the bone marrow (BM) [1]. Mature MCs are found in most vascularized organs, surrounding blood vessels and nerves [2]. MC and their progenitors express the receptor for stem cell factor (SCF), KIT (CD117), a transmembrane type III tyrosine kinase receptor (RTK) [3]. Binding of SCF to KIT governs most if not all the aspects of human MC biology, from proliferation to differentiation, migration, activation and survival [4, 5].

Mastocytosis are a heterogeneous group of diseases characterized by accumulation of abnormal (neoplastic) MCs in one or several organs, affecting both children and adults [6]. In adults, most patients present with a systemic involvement (systemic mastocytosis; SM). According to the World Health Organization (WHO), SM is classified into four major categories. Indolent SM (ISM) has a good prognosis, usually requires only symptomatic therapies, and ISM patients have a nearly normal life expectancy [6, 7]. The three other categories, collectively termed advanced SM, share a poor prognostic. Advanced SM categories include SM with an associated hematologic neoplasm (SM-AHN), aggressive SM (ASM), and MC leukemia (MCL) [8]. All categories of SM are characterized by an accumulation of abnormal MCs in BM and in other extra-cutaneous organs [9, 10].

WHO criteria for the diagnostic of SM include one major criterion and four minor criteria. The diagnosis of SM is established if at least the major and one minor criterion or at least three minor criteria are fulfilled [8, 11]. The major diagnostic criterion is defined by the presence of aggregates of at least 15 MCs identified by tryptase staining in BM and/or other extra-cutaneous organ biopsies. Minor criterions are: i) presence of 25% MCs with atypical morphology in BM smears [12], ii) aberrant immunophenotype of MCs (expression of CD2 and/or CD25) [13], iii) presence of an activating point mutation in codon 816 of the *KIT* gene in BM, peripheral blood (PB) or other extracutaneous organs [14, 15], and iv) increased level of serum tryptase (> 20 ng/mL) [16]. If SM is diagnosed, the next step is to evaluate its aggressiveness according to the presence of B-findings (borderline benign) and C-finding (consider cytoreduction). B- and C-findings correspond respectively to high MC burden and to organ dysfunction leading to the use of cytoreductive therapy [7]. The absence of B- and C-findings reflects an ISM [6, 7]. By contrast, the presence of at least one C-findings directs the diagnosis towards an advanced form of SM (ASM, SM-AHN or MCL). ASM is diagnosed when MCs in BM smears represent less than 20% of total nucleated cells [8, 17]. MCL is diagnosed when MCs in BM smears represent > 20% of total nucleated cells (with or without circulating neoplastic MCs in the bloodstream) [8, 17].

*KIT* is crucially involved in the pathophysiology of SM as the majority of patients carry *KIT* mutations, notably *KIT* D816V [18]. This mutation, found in > 85% of all patients with SM, activates constitutively KIT [15]. Activated KIT induces sustained proliferative and anti-apoptotic signaling in neoplastic MCs [18]. This KIT mutant receptor is resistant to most type I tyrosine kinase inhibitors (TKIs) targeting the wild-type receptor (KIT WT), such as imatinib [19]. By contrast, the mutant is sensitive to multikinase inhibitors such as midostaurin (PKC412) [20]. Thus, midostaurin is currently under clinical trials to treat Advanced SM [21]. However, this drug seems not sufficient to induce long-lasting complete responses in ASM and MCL [21]. There is thus a need to identify novel therapies for these diseases. To this purpose, relevant preclinical *in vivo* models of SM may be very useful. Unfortunately, the present lack of *in vivo* models mimicking at the best human *KIT* D816V^+^ advanced SM hampers pathophysiological studies and the development of new therapeutics. Only a few mouse models have been previously developed. In 2005, Zappulla *et al.* described a transgenic murine model using the primate chymase promoter as a driver of human *KIT* D816V mutation [22]. The authors reported the development of a SM-like disease within 12 to 24 months in only 30% of transgenic mice [22]. A few years after, Gerbaulet *et al.* described another transgenic mouse model expressing *kit* D814V mutation (murine homolog to human *KIT* D816V) [23]. However, this model has a limited utility because of the variety of diseases observed, ranging from perinatal lethality to pure MC hyperplasia, and of a very long period of latency (52 weeks) [23]. Besides, two xenograft mouse models have been described: one consisted in the injection of the leukemic HMC-1.2 cell line in SCID mice, giving rise to solid tumors [24], while the second consisted in injecting P815 mastocytoma cells in DBA-2 mice [25]. In this latter model, a severe mortality was observed within 9 days in injected animals [25]. These models are not useful for preclinical studies because of drawbacks such as the low incidence of disease and/or the kinetic of disease appearance and progression, which is either very short or too long. Thus, to date, no relevant *in vivo* models of *KIT* D816V^+^ advanced SM are available. In addition, none of the existing models allows monitoring of disease progression in real time.

We have recently reported on a new tumorigenic *KIT* D816V^+^ human MC line, termed ROSA^KIT D816V^, which presents most of the characteristics of neoplastic MCs found in ASM/MCL [26]. ROSA^KIT D816V^ cell line was obtained by stable lentiviral transfection of a construct encoding for the *KIT* D816V mutant gene in the parental, non tumorigenic human mast cell line, ROSA *KIT* wild-type (ROSA^KIT WT^) [26]. In the present study, we have stably transduced ROSA^KIT D816V^ cells with a construct encoding the naturally secreted *Gaussia princeps* luciferase (Gluc). This allowed us to obtain a new human neoplastic MC line, termed ROSA^KIT D816V-Gluc^. Gluc reporter is a highly sensitive luciferase allowing simultaneously the quantification of cells engraftment by measuring its activity in peripheral blood (PB) and *in vivo* imaging system (IVIS) [27]. ROSA^KIT D816V-Gluc^ cells were then injected in NOD-SCID IL-2R γ^−/−^ (NSG) mice, giving rise to a unique humanized *in vivo* model of *KIT* D816V^+^ advanced SM. In this model, disease progression and localization of neoplastic MCs were monitored *in vivo* and *ex vivo* using Gluc as a reporter [27].

## RESULTS

### Generation and characterization of ROSA^KIT D816V-Gluc^ cells secreting luciferase

**Figure 1:**
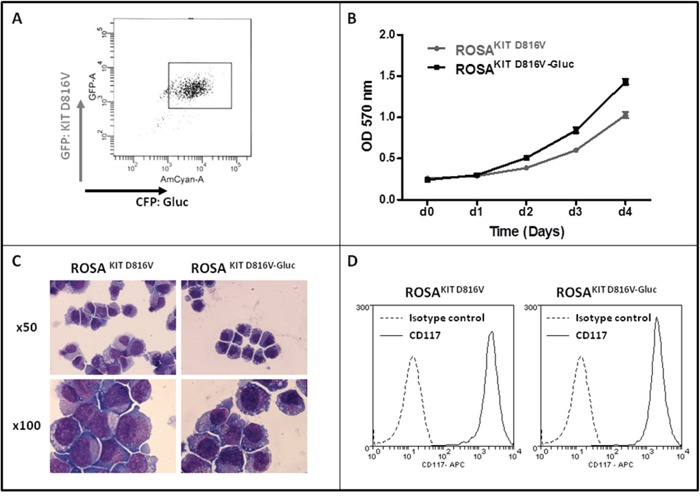
Generation of ROSA^KIT D816V-Gluc^ cell line and comparison with the parental ROSA^KIT D816V^ cell line **A.** ROSA^KIT D816V-Gluc^ GFP^+^/CFP^+^ sorted cells using flow cytometry. **B.** Comparison of ROSA^KIT D816V^ and ROSA^KIT D816V-Gluc^ cell proliferation by the use of the MTT method. Cells were seeded in 96-well plate for 5 days (1 plate/day). At each day (d0 - d4), 20 μL of MTT were added in each well and the cells were incubated for 3 additional hours at 37°C. After adding 100 μL of acidified isopropanol, the number of living cells was measured for each condition by reading the absorbance at 570 nm. Data are presented as the mean ± SD (n = 3). **C.** Phenotypic comparison of the cytological aspect of ROSA^KIT D816V^ and ROSA^KIT D816V-Gluc^. MGG-stained cytospin preparations of ROSA^KIT D816V^ (left panel) and ROSA^KIT D816V-Gluc^ (right panel). Magnification is x50 (top), x100 (bottom). **D.** Comparative expression of CD117 (KIT) by in ROSA^KIT D816V^ (left) and ROSA^KIT D816V-Gluc^ (right) analyzed by flow cytometry.

**Figure 2:**
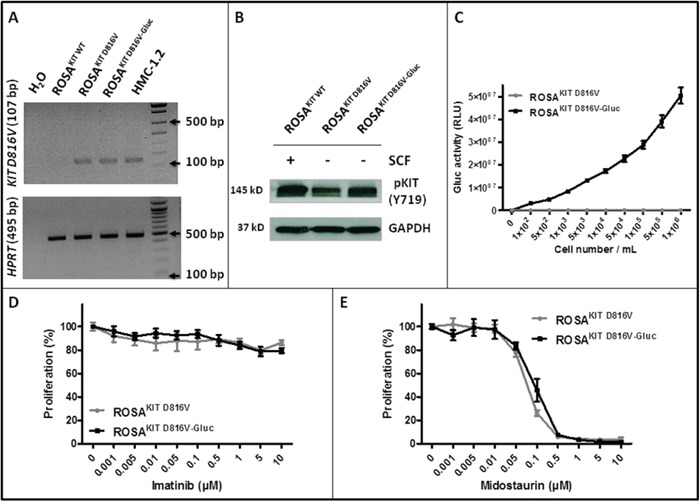
Effects of transduction of ROSA^KIT D816V^ cells with *LV-Gluc-CFP* on KIT activation and response to TKIs **A.** Analysis of *KIT* D816V gene expression in ROSA^KIT D816V-Gluc^ cell line using agarose gel electrophoresis of conventional RT-PCR products. Reactions were compared to the levels of expression of mRNA for HPRT. The image is an inverted form of the original picture. **B.** Detection of spontaneous phosphorylation of KIT receptor by western blotting in ROSA^KIT D816V-Gluc^. Cell lysates from ROSA^KIT WT^ cells stimulated with SCF, or from unstimulated ROSA^KIT D816V^ and ROSA^KIT D816V-Gluc^ cells, were subjected to electrophoresis and stained with antibody against p-KIT Y719. An anti-human glyceraldehyde-3-phosphate dehydrogenase (GAPDH) was used as a loading control. The lanes were run on the same gel. **C.** Gluc activity in cell culture supernatants of ROSA^KIT D816V^ (grey line) and ROSA^KIT D816V-Gluc^ (black line). Signals were calculated as RLU; relative luminescent units. Data present the mean ± SD (n = 3). **D, E.** Effect of Imatinib or Midostaurin on the proliferation of ROSA^KIT D816V^ (grey line) and ROSA^KIT D816V-Gluc^ cells (black line). Each cell lines were seeded for 72h in 96-well plate in the presence of various concentrations (0.001 - 10 μM) of Imatinib (D) or Midostaurin (E). Data are presented as the mean ± SD (n = 3) and are expressed as percent of proliferation in each condition relative to the control (untreated cells) considered as 100% proliferation.

We transduced the human ROSA^KIT D816V^ MC line stably expressing the *KIT* D816V mutant and GFP [26] with a lentiviral vector expressing Gluc and CFP*, LV-Gluc-CFP*. Cells expressing Gluc (ROSA^KIT D816V-Gluc^ cells) were sorted by selection of GFP^+^/CFP^+^ cells (Figure 1A) and cultured in the same medium than their parental cell line. As shown in Figure 1B, the introduction of the lentiviral vector expressing Gluc and CFP into ROSA^KIT D816V^ cells did not induce a significant change in their proliferation rate. In addition, the transduction did not change the phenotypic characteristics of the cells, as evidenced by the similar morphology of ROSA^KIT D816V^ and ROSA^KIT D816V-Gluc^ cells when observed on cytospin preparation after May Grünwald Giemsa (MGG) staining (Figure 1C). In addition, both cell lines expressed equally KIT (CD117) (Figure 1D). Besides, allele specific RT-PCR experiments confirmed that the two MC lines expressed the *KIT* D816V mutant at the same level (Figure 2A). Of note, as already described for ROSA^KIT D816V^ cells [26], the KIT receptor was constitutively phosphorylated in ROSA^KIT D816V-Gluc^ cells in the absence of SCF (Figure 2B). This level of phosphorylation was comparable in the two *KIT* D816V^+^ MC lines, and slightly higher in ROSA^KIT WT^ cells stimulated by SCF (Figure 2B). We then determined the level of Gluc activity in the cell-free supernatants of both *KIT* D816V^+^ cell lines cultured at various concentrations, using the Gluc substrate coelenterazine (CTZ). Gluc acitivity was detected at a significant level in ROSA^KIT D816V-Gluc^ cells supernatants after 24h of incubation, even at low cell concentrations (10^2^ cells per mL). As expected, there was no Gluc activity detectable in ROSA^KIT D816V^ cell supernatants, at any cell concentration (Figure 2C). Moreover, we observed that increasing the number of ROSA^KIT D816V-Gluc^ cells increased Gluc activity in a virtually linear manner (Figure 2C). Finally, in order to demonstrate that the transduction of by *LV-Gluc-CFP* did not change the sensitivity of the cells to TKIs, we assessed the effects of Imatinib or of Midostaurin on the proliferation of both *KIT* D816V^+^ cell lines. As expected, both cell lines were equally resistant to Imatinib (Figure 2D) and similarly sensitive to the antiproliferative effects of Midostaurin. The IC_50_ for Midostaurin was found at 70 nM for ROSA^KIT D816V^ cells and at 94 nM for ROSA^KIT D816V-Gluc^ cells (Figure 2E).

### Analysis of the level of engraftment of ROSA^KIT D816V-Gluc^ cells *in vivo* in NSG mice

**Figure 3:**
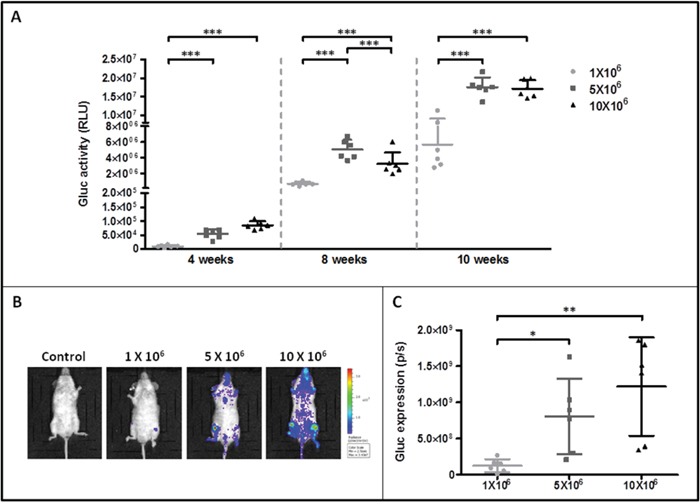
Monitoring of ROSA^KIT D816V-Gluc^ cells engraftment in injected mice by measurement of Gluc activity in blood and by IVIS **A.** Gluc activity in plasma of mice after various times of engraftment. Different numbers of ROSA^KIT D816V-Gluc^ cells [1x10^6^ (●), 5x10^6^ (■) and 10x10^6^ (▲)] were injected intravenously in mice and engraftment was monitored over time (4, 8 and 10 weeks). Each point represents an individual mouse. **B.** IVIS showing localization of Gluc in injected mice after 10 weeks. Indicated numbers of ROSA^KIT D816V-Gluc^ cells were IV injected in mice (n = 6). Units in rainbow color scales are photons per second per cm^2^ per steradian (p/sec/cm^2^/sr). Results shown are from one representative mouse for each group. **C.** Total relative units (RLU) per second were calculated for Gluc intensity shown in (B) by ROI analysis after 10 weeks of engraftment in the three groups of mice. Each point represents an individual mouse.

Various amounts of ROSA^KIT D816V-Gluc^ cells were injected intravenously in irradiated NSG mice. We then measured Gluc activity in the plasma of grafted mouse four, eight and ten weeks after cell injection. Interestingly, at 4 weeks of engraftment, the Gluc activity in plasma was found linearly increased in relationship with increasing numbers of injected cells (R^2^ = 0.97) (Figure 3A). However, although Gluc activity increased in a time-dependent manner, there was no apparent difference between groups injected with 5x10^6^ or 10x10^6^ cells at 8 and 10 weeks (Figure 3A). Compared with the level of Gluc activity determined in plasma, Gluc intensity measured after 10 weeks of engraftment by IVIS was found heterogeneous among groups, particularly in the groups injected with 5x10^6^ or 10x10^6^ cells at 10 weeks (Figure 3B and 3C).

**Figure 4:**
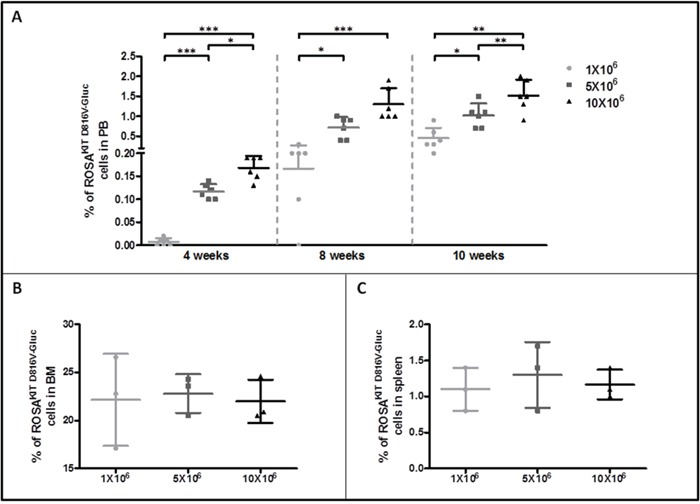
Evaluation of ROSA^KIT D816V-Gluc^ cells numbers by flow cytometry in peripheral blood, bone marrow and spleen **A.** Percentage of ROSA^KIT D816V-Gluc^ cells in PB after increasing times of engraftment (4, 8 and 10 weeks). The percentage of ROSA^KIT D816V-Gluc^ cells was determined in the three groups of mice: 1x10^6^ (●), 5x10^6^ (■) and 10x10^6^ (▲) by flow cytometry using monoclonal antibodies against human CD45 and human CD117. **B, C.** Percentages of ROSA^KIT D816V-Gluc^ cells after 10 weeks of engraftment in BM and spleen respectively in the three groups of mice: 1x10^6^ (●), 5x10^6^ (■) and 10x10^6^(▲). Each point represents an individual mouse.

To further investigate the disease progression in the three groups of xenografted mice, PB samples were analyzed for the presence of ROSA^KIT D816V-Gluc^ cells by quantifying the percentage of hCD45^+^/hCD117^+^ cells using flow cytometry. The percentage of ROSA^KIT D816V-Gluc^ cells in PB was found very low at 4 weeks (around 0.007 % in the 1x10^6^ cells group, 0.12 % in the 5x10^6^ cells group and 0.17% in the 10x10^6^ cells group). This percentage increased at 10 weeks in a dose (slightly > 0.45% in the 1x10^6^ cells group, > 1 % in the 5x10^6^ cells group and 1.5 % in the 10x10^6^ cells group at 10 weeks) and time-dependent manner (Figure 4A). The same phenomenon was observed when calculating the absolute numbers of ROSA^KIT D816V-Gluc^ cells in PB at different time periods and after injection of different numbers of cells (Table 1).

**Table 1:** Absolute numbers of ROSA^KIT D816V-Gluc^ cells in PB of NSG mice injected with different amounts of cells

	ROSA^KIT D816V-Gluc^ cell number in PB (x10^6^/mL)		
Groups	4 weeks	8 weeks	10 weeks
**1x10^6^ (n=6)**	0	0.006 ± 0.004	0.012 ± 0.004
**5x10^6^ (n=6)**	0.004 ± 0.001***	0.021 ± 0.007*	0.025 ± 0.009*
**10x10^6^ (n=6)**	0.003 ± 0.001***	0.042 ± 0.013***	0.039 ± 0.017*

Data are presented as mean ± SD and paired t-test was performed in comparison to the group receiving 1x10^6^ cells.

### Engraftment of ROSA^KIT D816V-Gluc^ cells *in vivo* in NSG mice leads to an advanced SM phenotype

To evaluate the level of ROSA^KIT D816V-Gluc^ cells engraftment in BM and spleen, thus the severity of the disease, mice (n=3 per group) were euthanized in each group 10 weeks after engraftment. BM cells were counted and the percentage of hCD45^+^/CD117^+^ cells was determined in each group. As expected, and in line with the human situation for ASM/MCL, the percentage of ROSA^KIT D816V-Gluc^ cells in BM was found much higher (around 22 %) than in spleen (1.1 %) and in PB (1.0 %). There was no difference in cell percentages between the three groups of mice in BM as well as in spleen (Figure 4B and 4C, respectively) and this was confirmed by calculation of the absolute number of ROSA^KIT D816V-Gluc^ cells in affected tissues (Table 2). Interestingly, the percentage of ROSA^KIT D816V-Gluc^ cells in BM reached a plateau at 10 weeks regardless the number of ROSA^KIT D816V-Gluc^ cells injected (Figure 4B and Table 2). All in all, the high percentages of neoplastic MCs found in BM, together with a moderate infiltration of the spleen and of the PB, might indicate that diseased animals suffer of a pathology similar to an aleukemic MCL, as observed in some human patients [28].

**Table 2:** Absolute numbers of ROSA^KIT D816V-Gluc^ cells in affected tissues of NSG mice injected with different amounts of cells after 10 weeks of engraftment

	ROSA^KIT D816V-Gluc^ cell number (x10^6^/mL)		
Groups	PB	Spleen	BM
**1x10^6^ (n=3)**	0.012 ± 0.004	0.856 ± 0.13	4.509 ± 0.690
**5x10^6^ (n=3)**	0.025 ± 0.009*	1.091 ± 0.294	3.618 ± 0.669
**10x10^6^ (n=3)**	0.032 ± 0.007*	1.134 ± 0	4.323 ± 0.802

Data are presented as mean ± SD and paired t-test was performed in comparison to the group receiving 1x10^6^ cells.

**Figure 5:**
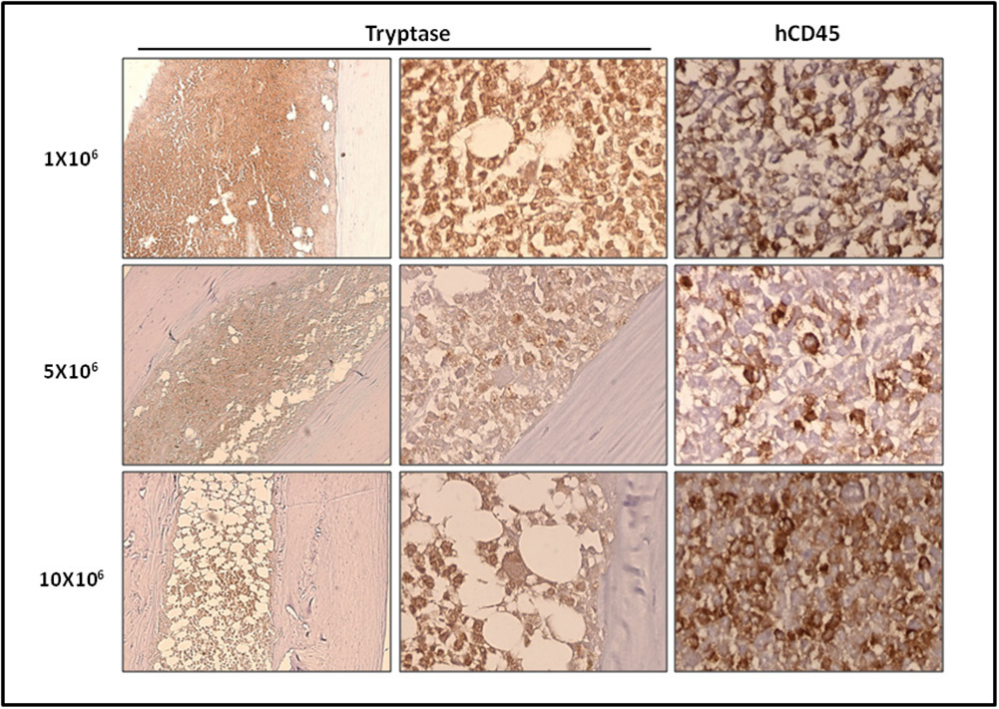
Localization of primary engrafted ROSA^KIT D816V-Gluc^ cells in mice BM by IHC detection of tryptase and hCD45 Ten weeks after engraftment, BM sections from the three groups of mice were stained by IHC with an anti-human tryptase antibody (left and middle panels) antibody and an anti-human CD45 antibody (right panel). Staining was visualized by Histomouse Kit, showing human MCs in brownish staining. Magnification is x10 (left panel) and x50 (middle and right panels). Results are from one representative mouse from each group.

**Figure 6:**
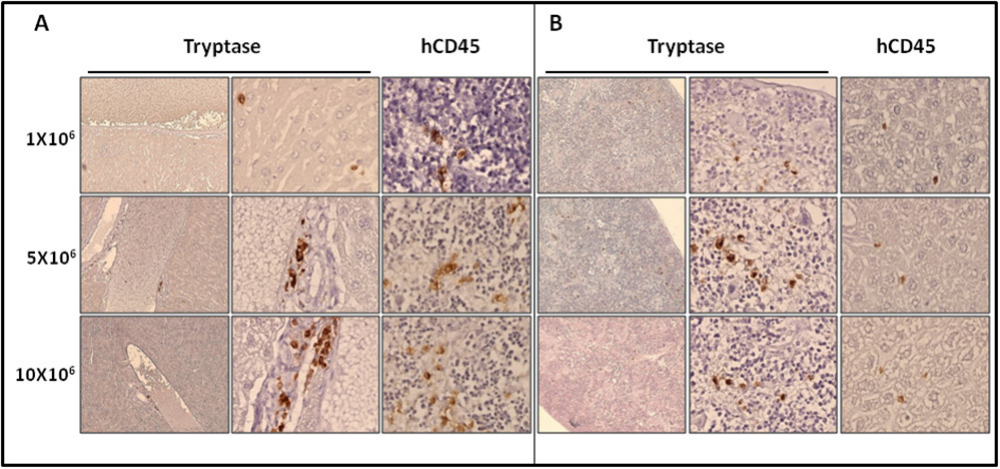
Localization of primary engrafted ROSA^KIT D816V-Gluc^ cells in mice spleen and liver by IHC detection of tryptase and hCD45 Ten weeks after engraftment, spleen **A.** and liver **B.** sections from the three groups of mice were stained by IHC with an anti-human tryptase (left and middle panels) antibody and an anti-human CD45 (right panel) showing human MCs in brownish staining. Magnification is x10 (left panel) and x50 (middle and right panels). Results are from one representative mouse from each group.

We then studied in detail the organs engrafted by ROSA^KIT D816V-Gluc^ cells, using immunohistochemical (IHC) staining with antibodies directed against human tryptase and human CD45. Analysis of BM sections of mice euthanized at 10 weeks revealed the presence of fibrosis, especially in the group injected with 10x10^6^ cells, as well as the presence of large clusters of tryptase and hCD45 positive cells (Figure 5), consistent with the pathological appearance of BM biopsies in human patients with advanced SM [29]. Whatsoever, tryptase^+^ and hCD45^+^ cells were detected at a high level in BM sections but in a lower extent in spleen (Figure 6A) and liver sections (Figure 6B). In the last two organs, a few clusters of MCs and isolated MCs were observed in all the organ sections (Figure 6A and 6B).

To further confirm the relevance of our *in vivo* model of SM towards the human pathology, we measured serum human tryptase levels in PB of xenotransplanted animals at 10 weeks. Consistent with the observation that tryptase serum levels are elevated in SM patients [30], significant levels of human tryptase (ranging from > 7.0 to > 11.0 μg/L) were measured in all xenotransplanted mice compared to the negative control, where the level of tryptase was found undetectable (< 1 μg/L) (Table 3). Interestingly, increased levels of serum tryptase paralleled increased numbers of cells injected at the beginning of the experiments (Table 3).

**Table 3:** Serum tryptase levels in NSG mice injected with different numbers of ROSA^KIT D816V-Gluc^ cells

Groups	Tryptase level (μg/L)
**Negative control**	< 1.00
**1x10^6^ (n=3)**	7.29 ± 1.00
**5x10^6^ (n=3)**	9.17 ± 0.31*
**10x10^6^ (n=3)**	11.65 ± 2.56

Data are presented as mean ± SD and paired t-test was performed in comparison to the group receiving 1x10^6^ cells.

Finally, after 12 weeks of transplantation, signs of health deterioration appeared in mice injected respectively with 5x10^6^ or 10x10^6^ ROSA^KIT D816V-Gluc^ cells, such as a decrease in water and food consumption accompanied with bristly hair, prostration and paralysis. This clinical phenotype further confirmed that the mice suffered from an aggressive form of the disease.

### Xeno-transplanted ROSA^KIT D816V-Gluc^ cells have enhanced grafting capabilities

**Figure 7:**
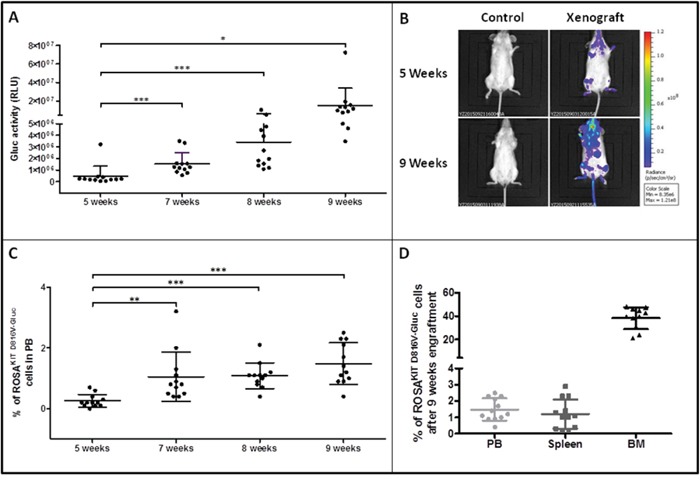
Reconstitution capacity of the human ROSA^KIT D816V-Gluc^ cells in NSG mice after secondary transplantation Ten weeks after engraftment in primary recipient mice, human cells were isolated from BM samples and injected into secondary recipient mice (n = 12). Engraftment was assessed 5, 7, 8 and 9 weeks after injection. **A.** Time-dependent increase of Gluc activity in plasma after secondary transplantation with ROSA^KIT D816V-Gluc^ cells. Each point represents an individual mouse. **B.** *In vivo* imaging of Gluc activity in secondary injected mice (right lane) after 5 weeks (top lane) and 9 weeks (bottom lane) versus negative control (left lane). Results shown are from one representative mouse. **C.** Time-dependent increase in the percentages of ROSA^KIT D816V-Gluc^ cells in PB after secondary transplantation. **D.** Percentage of ROSA^KIT D816V-Gluc^ cells after secondary transplantation in PB (●), spleen (■) and BM (▲). The percentage of hCD45^+^/hCD117^+^ was measured using flow cytometry in C and D.

In order to appreciate if ROSA^KIT D816V-Gluc^ cells were able to retain their grafting capabilities in a secondary transplant model, we grafted naive irradiated animals with a low number of human cells purified from the BM of previously xenotransplanted mice. Five weeks after the secondary transplantation, the efficacy of engraftment was assessed by measurement of Gluc activity in plasma, assessment of whole body by IVIS, and by measurement of the percentage of hCD45^+^/hCD117^+^ cells in PB. As previously seen in the experiments of first transplantation, we observed that Gluc activity increased in a time-dependent manner in the plasma (Figure 7A). In addition, as compared to non-injected control mouse, Gluc was detected by IVIS as soon as 5 weeks following the xenotransplantation in the majority of the mice injected with previously engrafted ROSA^KIT D816V-Gluc^ cells. The bioluminescence was found localized mainly in femur and feet (Figure 7B). Of note, after 9 weeks of secondary transplantation, Gluc expression was considerably increased when measured by IVIS (Figure 7B). Interestingly, in mice xenografted with pre-transplanted ROSA^KIT D816V-Gluc^ cells, the percentage of hCD45^+^/hCD117^+^ cells in PB, as well as the level of Gluc activity, were found greater after the secondary transplantation of 5x10^5^ cells than after the primary transplantation of 10x10^6^ cells, suggesting that the first transplantation selected more “mice adapted” neoplastic MCs.

**Figure 8:**
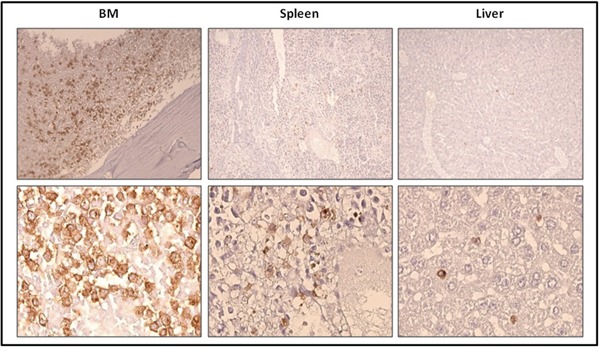
Localization of previously xenotransplanted ROSA^KIT D816V-Gluc^ cells in mice BM using IHC detection of hCD45 after secondary transplantation BM sections (left panel), spleen sections (middle panel), and liver sections (right panel) stained by IHC with anti-human CD45 antibody showing human cells in brownish staining. Magnification is x10 (top lane) and x50 (bottom lane). Each result shown is from one representative mouse having received previously xenotransplanted ROSA^KIT D816V-Gluc^ cells.

**Figure 9:**
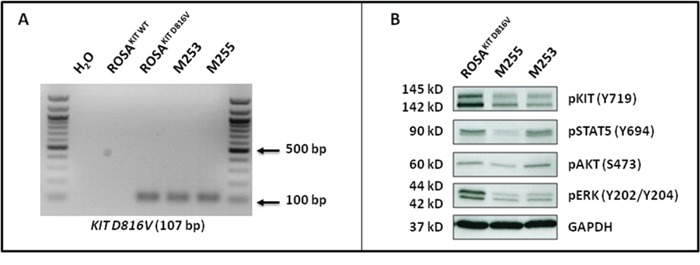
ROSA^KIT D816V-Gluc^ cells express *in vivo KIT* D816V mutant leading to constitutive phosphorylation of KIT and of downstream signaling pathways after secondary transplantation **A.** Expression of the *KIT* D816V gene in BM samples of injected mice using allele-specific RT-PCR. *KIT* D816V allele-specific PCR was performed using ROSA^KIT D816V^ cells as a positive control. The image is an inverted form of the original picture. **B.** Spontaneous phosphorylation of the KIT receptor and of its downstream signaling pathways (STAT5, AKT and ERK) revealed by western blotting in ROSA^KIT D816V^ cells and BM cells of secondary transplanted mice. An anti-human glyceraldehyde-3-phosphate dehydrogenase (GAPDH) was used as a loading control. The lanes were run on the same gel.

To evaluate more precisely the progression of the disease in the mice injected with previously transplanted cells, we measured the percentage of ROSA^KIT D816V-Gluc^ cells in PB, BM and spleen after 5, 7, 8 and 9 weeks of secondary transplantation through the quantification of hCD45^+^/hCD117^+^ cells by flow cytometry. We observed that the percentage of ROSA^KIT D816V-Gluc^ cells in PB increased significantly in a time-dependent manner (Figure 7C). Besides, at 9-weeks period of engraftment, the percentage of ROSA^KIT D816V-Gluc^ cells was found much higher in BM (around 35 %) than in spleen (around 1.5 %) or in PB (1.5 %) (Figure 7D). This observation was confirmed after IHC staining of human CD45 in BM, spleen, and liver (Figure 8). Finally, to confirm that secondary transplantation of the human *KIT* D816V^+^ MC line did not alter the expression of the mutant *KIT* gene, we explored the expression of *KIT* D816V mutant on BM cells of secondary transplanted mice using allele-specific RT-PCR. As shown on Figure 9A, BM cells of the two injected mice expressed the *KIT* D816V mutant, exactly as did the ROSA^KIT D816V^ cell line. As expected, no expression of the *KIT* mutant was detected in control, *KIT* mutant-negative ROSA^KIT WT^ cells (Figure 9A).

### Xenotransplanted ROSA^KIT D816V-Gluc^ cells have advanced SM-like alterations in signaling pathways

In *KIT* D816V^+^ neoplastic MCs found in the BM of SM patients, and particularly those with advanced SM, KIT is found spontaneously activated, but also other intracellular substrates are aberrantly activated (phosphorylated), such as signal transducer and activator of transcription 5 (STAT5), AKT and extracellular signal-regulated kinases 1/2 (ERK1/2) [31, 32]. Those signaling molecules have been found increasingly phosphorylated in parallel with the aggressiveness of the disease and resistance of the neoplastic MCs to TKIs [31, 32]. To further confirm the similarity of our *in vivo* model of *KIT* D816V^+^ advanced SM with the human situation, we analyzed the spontaneous phosphorylation state of KIT, STAT5, AKT and ERK in BM cells isolated from secondary injected mice, as compared to the ROSA^KIT D816V^ parental cell line. As shown in Figure 9B, the constitutive activation of the KIT receptor was accompanied by aberrant phosphorylation of KIT-downstream signaling pathways such as AKT, STAT5 and ERK. These findings are consistent with those reported for neoplastic MCs in advanced SM patients [31].

## DISCUSSION

SM are rare neoplasms characterized by a specific, recurrent and almost always acquired molecular defect inducing constitutive activation of the KIT receptor [33]. The most frequent defect found in all categories of SM is the *KIT* D816V point mutation [18]. *KIT* D816V mutant is found in > 90% of all ISM cases and in up to 80% of advanced SM patients [34]. Recently, we have established a unique human MC line, termed ROSA^KIT D816V^, which is considered as the first relevant *in vitro* model of human neoplastic MCs bearing only the *KIT* D816V mutant [26]. We reported that this *KIT* D816V^+^ MC line is highly tumorigenic in immunocompromised mice, by contrast to its parental cell line presenting with a wild type *KIT* (ROSA^KIT WT^) [26]. However, to the best of our knowledge, there is no *in vivo* model of engraftment of human *KIT* D816V^+^ MCs where of the disease progression can be monitored repeatedly by a simple, non-stressing technique. Thus, we took advantage of the availability of our ROSA^KIT D816V^ cell line to generate a new *in vivo* model of engraftment of human *KIT* D816V^+^ neoplastic MCs in NSG mice, using the Gluc reporter system, that allows to monitor the disease progression by a simple measurement in blood samples, in real time, without stressing the animals [27, 35].

In the first step of our work, we generated ROSA^KIT D816V^ cells expressing the secreted *Gaussia princeps* luciferase [27, 35], termed ROSA^KIT D816V-Gluc^ cells. We then confirmed by the measurement of Gluc activity in the cell supernatants that ROSA^KIT D816V-Gluc^ cells effectively express Gluc, as previously reported for other cell models [27]. In addition, we demonstrated that lentiviral infection of ROSA^KIT D816V^ cells with the Gluc-containing construct did not alter their morphology, the *KIT* D816V expression, the constitutive phosphorylation of the KIT receptor, and their SCF-independence. In addition, we demonstrated that the ROSA^KIT D816V-Gluc^ cells retained the same sensitivity (or resistance) to different TKIs than the parental ROSA^KIT D816V^ cell line, making it a very attractive tool to screen for new targeted drugs *in vitro* and *in vivo*. Particularly, as previously described in other neoplastic MC models [20], we confirmed the resistance of our *KIT* D816V^+^ MC model towards Imatinib, while the cells remained sensitive to Midostaurin.

With the aim to establish a new *in vivo* model of *KIT* D816V^+^ SM in which the progression of the disease could be easy to follow accurately, we injected various numbers of ROSA^KIT D816V-Gluc^ cells in previously irradiated NSG mice. Expression of Gluc by injected cells allowed us to check and monitor accurately not only the cell engraftment but also the progression of SM in NSG mice. Indeed, we observed that, after 4 weeks following the cell transplantation, Gluc activity in the PB of xenografted animals displayed a linear relationship with the number of cells injected, as previously described for other *in vivo* models [27]. However, in our study, there was no apparent difference between the 5x10^6^ and the 10x10^6^ groups at 8 and 10 weeks, suggesting that over a given number of cells injected, saturation is reached (Figure 3A). Since Gluc blood assay complements IVIS, which has the ability to localize the signal in organs [36], we analyzed Gluc expression in the whole body of the injected mice. Quantification through ROI analysis revealed that Gluc intensity was heterogeneous among groups. Comparing the two methods, measurement of Gluc activity in plasma seemed thus more reliable than measurement of Gluc intensity by IVIS to reflect the total MC burden in the animals. In conclusion, Gluc assay in PB provides both a sensitive and quantitative assessment of engraftment, complementing IVIS, which has the ability to localize the signal (thus the grafted cells) in different organs of the animals.

Furthermore, we showed that ROSA^KIT D816V-Gluc^ cells can engraft all injected NSG mice, not only at the BM level but also in spleen, liver and PB, giving rise to a SM-like disease nearing the one found in humans, according to the WHO criteria for the diagnosis of SM [37]. The percentage of ROSA^KIT D816V-Gluc^ cells increased in a time- and number of cells injected-dependent manner, suggesting that the disease progression can be accelerated or slowed by varying the number of cells injected. As expected, the percentage of ROSA^KIT D816V-Gluc^ cells in BM was found much higher than in spleen and PB, suggesting that ROSA^KIT D816V-Gluc^ mainly accumulated in BM, as evidenced on BM sections stained for hMC tryptase and hCD45. However, the percentage of ROSA^KIT D816V-Gluc^ cells in BM was not strictly parallel to the increase of total ROSA^KIT D816V-Gluc^ cell number in mice, meaning that the cell number reached a plateau in the BM, while the cells continued to proliferate in other tissues. Whatsoever, the percentage of neoplastic human MCs reached 20% or above in the BM of injected animals. This criterion is diagnostic for MCL [8]. In addition, all injected mice presented a high level of serum tryptase (ranging from > 7.0 ng/mL for mice injected with 1x10^6^ cells to > 11.0 ng/mL for mice injected with 10x10^6^ cells), although not exceeding the threshold of 20 ng/mL, a value established for SM diagnosis in humans [8]. This may be explained by the relativity low content of tryptase of the ROSA^KIT D816V-Gluc^ cells as compared to neoplastic MCs found in the BM of SM patients (data not shown). In addition, one has to keep in mind that, contrasting to the human situation where tryptase is already detectable at several ng/mL in the bloodstream of healthy individuals, the level of human tryptase in healthy mice is equal to zero. Thus, any increase in human tryptase level in PB of our mice reflects MCs engraftment. Interestingly, the level of tryptase in plasma was significantly correlated with the level of Gluc in plasma at 10 weeks (p value = 0.007 - R square = 0.67), indicating that measuring Gluc activity in plasma is a reliable method to monitor disease progression in our *in vivo* model. Of note, while quantification of serum tryptase level required more than 50 μL of plasma, only 5 μL of blood or plasma samples are needed to measure Gluc activity.

All in all, our *in vivo* model of primary xenotransplantation fits well with the one of an advanced (ASM/MCL-like) SM disease, a fact further confirmed by the rapid deterioration of the health status of the mice after 12 weeks of engraftment. In line with this observation, it has to be underlined that the mean overall survival of MCL patients is usually very short in the absence of treatment, being of 6 months or less [38].

In addition, we have demonstrated that the transplantation of ROSA^KIT D816V-Gluc^ cells from primary engrafted NSG mice into secondary recipient increased their capacity of engraftment. This indicates that ROSA^KIT D816V-Gluc^ cells were adapted to the mouse microenvironment upon the first transplantation, explaining thus the acceleration of secondary engraftment. Such phenomenon has been previously shown in serial transplantations of other types of leukemic cells isolated from patients [39]. Interestingly, this enhanced engraftment capability of previously xenotransplanted ROSA^KIT D816V-Gluc^ cells might enable us to use these cells to achieve larger lots of mice for preclinical studies.

Of note, the *in vivo* model presented here is a unique model paralleling human *KIT* D816V^+^ advanced SM disease, with many advantages over previously published *in vivo* models which did not really reproduce the clinical and biological characteristics of advanced SM. In fact, injection of the human leukemic MC line HMC-1.2 exhibiting two *KIT* mutations, *KIT* V560G and *KIT* D816V - which is unusual in SM patients - in SCID mice, gave rise to solid tumors in 6 weeks to 5 months [24]. More recently, we have described a model of mice transgenic for *KIT* D816V, which expressed the human *KIT* D816V mutant specifically in MCs under the control of baboon chymase promoter [22]. However, only one third of the transgenic mice developed an ISM-like disease, with a moderate MC hyperplasia, after 12 to 24 months of latency [22]. In this model, the low disease penetrance and the delay of disease appearance made drug studies problematic, in addition to the fact that the *KIT* D816V mutant was expressed in murine MCs. In 2006, Demehri *et al.* have developed a murine model based on IV injection of P815 cells, a mouse mastocytoma cell line harboring *kit* D814Y mutant (analogue to *KIT* D816Y mutant in human) in DBA/2 mice [25]. This model gave rise to an ASM/MCL-like disease after 6-9 days, with a severe mortality at 9 days, making *in vivo* drug studies impossible to perform in a so short time. More recently, another transgenic mice model have been developed by Gerbaulet *et al*. using the Cre/loxP recombination system allowing conditional expression of the *Kit* D814V mutant (murine homolog of human *KIT* D816V mutation) under the control of the *Kit* promoter. In this model, the development of a SM-like disease followed a slow kinetics (around 52 weeks), and mice developed colitis associated with mucosal MC accumulation [23]. Thus, this model is far from nearing human *KIT* D816V^+^ advanced SM as it deals with a *Kit* D814V mutant expressed in murine MCs [23].

Furthermore, compared to previous models of SM, our *in vivo* model nears at the best the biological characteristics of neoplastic MCs found *in vivo* in advanced SM patients. Particularly, the presence of *KIT* D816V mutant, together with the constitutive activation of KIT, but also of STAT5, AKT and ERK in these cells mimic the situation observed in such patients where STAT5, AKT and ERK have been found increasingly phosphorylated in parallel with the aggressiveness of the disease and its resistance to TKIs [31, 32]. Our model will thus help to test targeted therapies, alone or in combination, directed not only against the *KIT* D816V mutant but also against other oncogenic signaling involved in the progression of the disease and in the resistance to TKIs *in vivo*.

In conclusion, the secretion of luciferase (Gluc) by our newly generated human *KIT* D816V^+^ Gluc^+^ neoplastic MC line greatly facilitates the assessment of engraftment in mice and allows the accurate monitoring of disease progression. Moreover, although MC engraftment is observed within 4 weeks in 100% of injected mice, health deterioration is observed after 12 weeks or even later, depending on the number of cells injected, allowing thus i) to increase or decrease disease aggressiveness by increasing or decreasing the number of injected cells and ii) sufficient time between the development of the disease and its fatal outcome, which is a critical requisite for easy preclinical drug studies. Such additional studies are ongoing in our laboratory on this model.

## MATERIALS AND METHODS

### Cell culture

The human parental, SCF-dependent non-tumorigenic MC line, ROSA^KIT WT^ was cultured exactly as previously described, in the presence of 10% of supernatant of Chinese hamster ovary (CHO) cells transfected with the murine *SCF* gene, used as a source of SCF [26]. The human neoplastic, SCF-independent tumorigenic MC line ROSA^KIT D816V^ was obtained by stable lentiviral transduction of a construct encoding for the human *KIT* D816V gene and GFP in the parental ROSA^KIT WT^ cell line, as already described [26] and was cultured in the absence of SCF, exactly as previously described [26]. The human MCL cell line HMC-1.2 harboring both *KIT* V560G and *KIT* D816V mutations (kindly provided by Dr. J. H. Butterfield; Mayo Clinic, Rochester, MN) was cultured exactly as already described [40].

### Transduction of ROSA^KIT D816V^ cells with *LV-Gluc-CFP* construct

Lentivirus vector construction and production of *LV-Gluc-CFP* were performed as previously described [27, 35]. Briefly, the *Gaussia* luciferase cDNA was cloned into lentivirus vector under the control of the strong constitutive cytomegalovirus (CMV) promoter to produce *LV-Gluc*. In another vector, cDNA for *Gluc* and the optimized *cyan fluorescent protein* (CFP) separated by IRES (*LV-Gluc-CFP*) were cloned under the CMV promoter. Lentivirus particles were produced by transfection of 293T cells as previously described [27]. ROSA^KIT D816V^ cells were then transduced overnight with lentiviral particles of *LV-Gluc-CFP* in order to produce ROSA^KIT D816V-Gluc^ CFP^+^ cells. The efficacy of transduction was measured after 7 days by flow cytometry. ROSA^KIT D816V-Gluc^ cells expressing both GFP and CFP were then sorted using a BD FACSAria (Beckton Dickinson Biosciences).

### Morphological and phenotypical analysis of the ROSA^KIT D816V-Gluc^ cell line

In order to ensure that lentiviral transduction of ROSA^KIT D816V^ cells with *LV-Gluc-CFP* construct have not affected the morphology of the cells, May-Grünwald Giemsa (MGG)-stained cytospin preparations of ROSA^KIT D816V^ and ROSA^KIT D816V-Gluc^ cells were examined using a Zeiss Axiophot microscope (Carl Zeiss). In addition, the expression of KIT (CD117) was analyzed on both cell lines cells by direct immunofluorescence using a FACSCalibur (BD Biosciences). Briefly, 2x10^5^ untreated cells were incubated with APC (allophycocyanin)-labeled monoclonal antibody directed against human CD117 (BioLegend). Isotype control was used for each experiment, and at least 10,000 events were recorded on a BD FACSCalibur.

### Allele specific RT-PCR for the detection of the *KIT* D816V mutant

Total RNA from 5x10^6^ ROSA^KIT WT^ cells, ROSA^KIT D816V^ cells, ROSA^KIT D816V-Gluc^ cells, HMC1.2 cells, or from 5x10^5^ human cells purified from BM of mice previously xenografted with ROSA^KIT D816V-Gluc^ cells was isolated using TRIzol (Life Technologies) or using Nucleospin RNA (Machery Nagel) according to the manufacturer's protocol. RNA concentration and purity was determined using a Nanovue plus spectrophotometer (GE Healthcare). Starting from these RNA samples, first strand cDNA synthesis was conducted using the iScript Select cDNA synthesis kit (Biorad) or using Superscript II reverse transcriptase (Invitrogen) according to the manufacturer's instructions. PCR was performed using primer sequences designed as described by Kristensen *et al.* [41]: a forward primer 5′-AGAGACTTGFCAGCCAGAAAA-3′, and a reverse primer 5′-TTAACCACATAATTAGAATCATTCTTGATCA-3′ for *KIT* D816V. Reactions were compared to levels of the hypoxanthine phospho-ribo-transferase (HPRT) amplified using the following primers: HPRT F; 5′-ATGGACAGGACTGAACGTCTTGC-3′; and HPRT R; 5′-GACACAAACATGATTCAAATCCCTGA-3′. Complementary DNA were amplified using the following cycle conditions: 95°C for 10 min, followed by 40 cycles of 95°C for 15 s, 60°C for 1 min and 72°C for 1 min. All PCR experiments included a no template control (water). The amplified PCR products were visualized by staining with ethidium bromide (Electran) after 1.5 % agarose gel electrophoresis in Tris–acetate–EDTA buffer (Euromedex).

### Western blot analysis for KIT signaling

Western blots were performed on cell lysates from ROSA^KIT WT^ cells stimulated with SCF, or from unstimulated ROSA^KIT D816V^ and ROSA^KIT D816V-Gluc^ cells, using an antibody against phosphorylated KIT Y719 (Cell Signaling Technology), in order to visualize the phosphorylation state of KIT. Western blots were also performed on lysates of mouse BM cells using antibodies against phosphorylated KIT Y719, phosphorylated STAT5 Y694, phosphorylated AKT S473 and phosphorylated ERK Y202/204 (all from Cell Signaling Technology). Proteins were visualized with horseradish peroxidase-conjugated secondary antibodes and chemoluminescent substrate (Promega). A mouse monoclonal IgG1 anti-human glyceraldehyde-3-phosphate dehydrogenase (GAPDH) Ab (Santa Cruz Biotechnology) was used as a loading control.

### *In vitro* gluc activity assay

To measure Gluc activity in cell supernatant, ROSA^KIT D816V^ cells expressing or not Gluc were seeded in 24-well plates at various concentrations (from 100 to 1x10^6^ cells/mL) and incubated for 24 hours at 37°C and 5 % CO_2_. Twenty microliters of conditioned medium were assayed using 100 μL (5 μg/mL) water-soluble coelenterazine (CTZ) (Nanolight Technologies) in full white plates. Photon counts were acquired immediately using a luminometer (PerkinElmer - EnSpire®).

### Analysis of the effects of TKIs on cell proliferation

To ensure that lentiviral transduction of ROSA^KIT D816V^ cells with *LV-Gluc-CFP* construct has not influenced the sensitivity of the cells towards different TKIs, the effect of Imatinib (Sequoia Research) and of Midostaurin (Novartis Pharmaceutical Corporation) on cell proliferation was measured using the 3-(4, 5-dimethylthiazol-2-yl)-2,5-diphenyl tetrazolium bromide (MTT) test. ROSA^KIT D816V^ and ROSA^KIT D816V^^−Gluc^ cells were incubated with Imatinib or Midostaurin (1 nM to 10 μM) in 96 well plates (Falcon) at 37°C for 72 hours. After incubation, 20 μl of MTT (5 mg/mL) (Life technologies) were added in each well and the plates were incubated for 3 additional hours at 37°C. Then, 100 μl of acidified isopropanol were added to each well to dissolve formazan crystals. The number of living cells was then measured in each condition by reading the absorbance at 570 nm using a plate reader (Thermo-Labsystems). Results were expressed in % of living cells as compared to the control (untreated cells).

### Xenogeneic transplantation of ROSA^KIT D816V-Gluc^ cells in NOD-SCID IL-2R γ^−/−^ (NSG) mice

NSG mice were bred and maintained under specific pathogen-free conditions at the animal facility of Gustave Roussy Institute. Animal experiments were performed in accordance with guidelines established by the Institutional Animal Committee.

Increasing amounts of ROSA^KIT D816V-Gluc^ cells (1x10^6^, 5x10^6^ or 10x10^6^) were injected to mice (3 groups; 6 mice per group) 24 h after irradiation at 2.5 Gy from a cesium-137 source. Engraftment was assessed after 4, 8 and 10 weeks using quantitative measurement of Gluc activity in plasma, and quantitative measurement of hCD45^+^/hCD117^+^ cells in PB using flow cytometry. After 10 weeks, *in vivo* bioluminescence imaging was performed on engrafted mice. *KIT* wild type (ROSA^KIT WT^ control cells) were not included in this study as a control because they hardly - if not at all - graft in mice, as described elsewhere [26].

### Secondary transplantations

Secondary transplantations were performed using human cells isolated from the BM of mice previously injected with ROSA^KIT D816V-Gluc^ cells. Briefly, after 10 weeks of engraftment, one femur and two tibias were recovered from each animal and flushed in 1 mL of PBS. BM cells were then purified using EasySep™ Mouse/Human Chimera Isolation Kit (Stem Cell) according to the manufacters' instructions. Purified BM cells were then pooled, and 5x10^5^ cells were IV injected to each NSG mouse (n = 12) 24 h after their irradiation at 2.5 Gy from a cesium-137 source.

### Flow cytometry analysis for the detection of xenografted cells

ROSA^KIT D816V-Gluc^ cells were quantified in mouse PB, BM and spleen samples by flow cytometry using PE-labeled monoclonal antibodies (mAb) directed against human CD117 (Beckman Coulter), PE-Cy7- labeled hCD45 and APC-labeled mCD45 (BD Biosciences). Engrafted ROSA^KIT D816V-Gluc^ cells were defined as hCD45^+^/CD117^+^ cells.

### Measurement of gluc activity *ex vivo* in peripheral blood

Blood samples were collected in EDTA pre-coated vials (KABE Labortecknik). Gluc activity was determined on 5 μl of plasma by adding 100 μl of water soluble CTZ (50 μg/mL) followed by immediate acquisition of photon counts using a luminometer (PerkinElmer - EnSpire) as previously described by Morse and Tannous [42].

### *In vivo* bioluminescence imaging

Mice were anesthetized using isoflurane and Gluc imaging was performed immediately after IV injection of 100 μl water soluble CTZ (4 mg/kg body weight) using a CCD camera (IVIS 50) and analyzed with Living Image® Software as previously described [42]. Quantitative analysis of Gluc intensity was performed by measuring luminescence signal intensity using region of interest (ROI) setting of the Living Image® software. ROIs were placed around the total area of body mice. ROI measurements were expressed as total flux (p/s).

### Histopathology and immunohistochemistry (IHC) analysis

Deparaffinized sections of BM, spleen and liver processed for heat-induced antigen retrieval were incubated with a mouse anti-human CD45 mAb (Dako) or with a mouse anti-human MC tryptase (Dako). Staining was visualized by Histomouse Kit (Zymed). The sections were then counterstained with hematoxylin and examined using a Zeiss Axiophot microscope (Carl Zeiss).

### Serum tryptase measurement

Total serum tryptase was measured using fluorescent enzyme-linked immunoassay that detects specifically human tryptase, following the manufacturer's recommendations (Unicap Pharmacia) [43]. The detection limit of this assay is 1 ng/mL, and in human healthy controls, serum tryptase levels range between less than 1 and 15 ng/mL, with a median of approximately 5 ng/mL [44].

### Statistical analysis

Statistical analysis was performed using GraphPad Prism 5.03 software. Data were analyzed using a two tailed Student's t test. The levels of significance were as follow: *, *p<0.05*; **, *p<0.01*; ***, *p<0.001*.
